# logLIRA: a method for reliable suppression of electrical stimulation artifacts enabling short-latency neural response recovery

**DOI:** 10.1088/1741-2552/ae5f4b

**Published:** 2026-05-12

**Authors:** Francesco Negri, David J Guggenmos, Federico Barban

**Affiliations:** 1 Department of Informatics, Bioengineering, Robotics, and Systems Engineering (DIBRIS), Università degli Studi di Genova, Genova, Italy; 2 Department of Physical Medicine and Rehabilitation, University of Kansas Medical Center, Kansas City, KS, United States of America; 3 IRCCS Azienda Ospedaliera Metropolitana, Genova, Italy

**Keywords:** ICMS, electrical stimulation, multielectrode array, artifact suppression, neural evoked activity

## Abstract

*Objective.* We propose logLIRA, a novel method for the rejection of intracortical microstimulation artifacts in electrophysiological recordings specialized in the recovery of short-latency evoked activity. Additionally, we introduce a comprehensive comparison framework to evaluate the performance of logLIRA against previously reported algorithms. *Approach.* Our method estimates the artifact profiles by means of a piece-wise linear interpolation between logarithmically distributed points. It handles signal saturation thanks to a dynamically adjusted blanking interval. Finally, it deals with residual secondary artifacts by clustering and common activity rejection. The artifact rejection proficiency of logLIRA is evaluated against other state-of-the-art algorithms by means of a semisynthetic dataset acting as ground truth and enabling the computation of key performance metrics. *Main results.* The benchmark analysis highlights that our new method outperforms its competitors, enabling a robust recovery of short-latency evoked activity and, at the same time, minimizing the likelihood of introducing false positives as a consequence of misclassified residual artifacts. The results hold for very heterogeneous artifact profiles (with or without signal saturation) and across different combinations of mean artifacts rate and mean firing rate in the semisynthetic dataset. Additionally, we show the functioning of logLIRA with real-world data, where its capability to handle secondary artifacts and avoid inflated evoked responses is particularly evident. *Significance.* This work provides the scientific community with a valuable and powerful tool to improve the recovery of evoked responses, potentially contributing to a better understanding of functional connectivity and brain plasticity mechanisms in *in vivo* neural circuits as well as the advancement of therapeutic electrical neuromodulation techniques.

## 1. Introduction

Neuromodulation is a key tool when it comes to study the basic mechanisms governing the interaction between neurons as well as understanding and treating a number of neuropathologies. The delivery of stimuli to elicit a response at the target cells level has historically been employed in a wide spectrum of scenarios, ranging from *in vitro* to *in vivo* experiments, even involving human participants [1–5]. Neural stimulation plays a pivotal role in a growing number of clinical contexts, with applications in the treatment of various neurological and neuropsychiatric disorders often through deep brain stimulation [4–6], the restoration of sensory perception in patients with disabilities [7–10], the provision of sensory feedback for neuroprostheses [11, 12], and the enhancement of plasticity to facilitate post-injury cortical rewiring [3, 13, 14].

In both fundamental research and clinical settings, electrical stimulation has long been the predominant neuromodulation technique. While it can take many forms, a common choice to precisely target small brain regions is intracortical microstimulation (ICMS), that involves the direct injection of small, localized electrical currents into cortical tissue using invasive miniaturized electrodes [5, 15, 16]. While electrophysiological recordings provide valuable insights into the correlation between neural circuitry and specific cognitive functions, electrical neuromodulation, particularly ICMS, offers notable potential to establish a causal link between specific brain regions and their associated functions [17–19].

Despite the significance of electrical neuromodulation to the scientific community, simultaneously stimulating and recording from nearby electrodes remains a complex challenge [1, 19–22]. When trying to understand the direct impact of electrical stimuli on the targeted circuitry, the recorded activity is hindered by large artifacts that mask neural firing patterns, as these injected voltage fluctuations are orders of magnitude larger than the extracellularly recorded action potentials [1, 18–21]. The stimulation artifacts not only overlap with the neural response, making it difficult to observe, but their high voltages often saturate the recording system amplifier or push it toward the edge of the operational range leading to nonlinear characteristics, preventing the recording of underlying activity [1, 20, 23]. These abrupt changes in voltage and subsequent rebounds arise from capacitive coupling and resistive crosstalk among the leads, surrounding tissue, and the recording device, as well as from leakage currents within the system [1, 20, 24–27]. The shape, duration, and magnitude of the artifacts are noticeably affected by a number of variables, including the electrical stimulus parameters, the stimulation protocol (i.e. the spatio-temporal pattern of multiple stimuli), the proximity between stimulation and recording sites, and properties related to the employed electrodes and acquisition systems [1, 21, 25].

While hardware solutions would ideally be preferable to avoid amplifier saturation or nonlinear operations, software algorithms for handling electrical stimulation artifacts generally offer greater versatility, scalability to high channel counts, and the ability to run on already acquired data, performing a *post hoc* suppression of stimulus artifacts [1, 18, 20, 21]. These offline methods span a wide range of techniques, from complete blanking (i.e. setting a certain number of samples immediately following the stimulus onset to placeholder values, such as zeros) to artifact template estimation and subtraction via averaging, as well as more sophisticated approaches [1, 28, 29].

One popular averaging-based method is dynamic averaging, an enhanced version of global averaging [1, 30, 31] that takes into account the variability of artifact waveforms by considering a sub-sample of temporally adjacent artifacts to estimate the template of each stimulus under the assumption that artifacts close in time exhibit more similar profiles [1, 32]. Other widely used methods often involve either local or global fitting of exponential and polynomial functions to estimate and later subtract artifact profiles [1, 20, 26, 33]. The main advantage of fitting techniques is their suitability for real-time operations, as they handle each artifact individually [20]. Another low-complexity strategy occasionally proposed is reverse or zero-phase filtering, either to estimate and subtract the artifact waveform or to directly isolate the superimposed neural activity. However, to prevent ringing, filtering methods require explicit handling of saturation and discontinuities, making them conceptually closer to fitting techniques than to a genuinely distinct class of artifact rejection methods. Notably, authors have previously compared local curve-fitting strategy (i.e. SALPA) against Butterworth and linear-phase filters, reporting poorer performance for those simpler filtering approaches in the recovery of evoked responses [20].

Although their use is widespread in literature, existing stimulus artifact rejection algorithms struggle to consistently handle electrical stimulation artifacts and recover short-latency evoked activity (i.e. spiking activity within 0.5–5 ms after the stimulation [34]), with mixed results across different electrophysiological recordings and electrical stimulation protocols. For example, dynamic averaging responds poorly to unevenly spaced stimulation pulses, due to the increased artifact profile variability. Artifact shape is in fact affected by both the absolute temporal occurrence of a stimulus and the relative distance from the previous pulse [20, 21]. Recently, researchers addressed this issue by grouping artifact waveforms according to their similarity via dictionary learning, but this approach is not suitable for recordings with saturation during the delivery of stimulation pulses [25]. Fitting techniques generally handle saturation and post-stimulus fast dynamics by introducing a longer blanking interval, resulting in the loss of signal and potentially precluding the observation of evoked activity [1, 18, 20, 21]. Forcing a shorter blanking interval introduces secondary artifacts, which can be misidentified as spikes if not properly handled.

Innovative algorithms leveraging on the shared structure of stimulus artifacts across adjacent electrodes have been proposed [18, 19, 35]. However, these approaches are limited by the requirement of dense neural tissue sampling [18, 19, 21], saturation-free recordings [18], or neural activity independent of electrical stimuli [21, 35]. This highlights the difficulty in realizing a generalizable solution for the suppression of stimulation artifacts, that performs well with different electrophysiological recordings, acquisition systems, and heterogeneous ICMS protocols.

In this paper, we introduce logLIRA (Logarithmic Linear Interpolation for the Removal of Artifacts), a novel algorithm for the rejection of stimulus artifacts that leverages on a piece-wise linear interpolation between logarithmically distributed points, denser in proximity to the stimulation pulse, where the artifact dynamics is faster. Our method is designed to successfully suppress considerably different artifact profiles, even in the presence of signal saturation or with an increased waveform variability due to unevenly spaced electrical pulses. Additionally, logLIRA exploits a secondary artifacts’ mitigation mechanism enabling the minimization of blanking periods and thus reducing signal loss, facilitating a reliable recovery of short-latency evoked activity.

To validate the capabilities of logLIRA, we developed a comprehensive comparison framework to evaluate its performance against previously reported algorithms, confirming its effectiveness. We generated a semisynthetic benchmark dataset from data recorded with various commercial systems during experimental sessions involving rodents and non-human primates, providing a reliable ground truth for comparison. This enabled us to thoroughly assess the algorithms’ ability to recover artifact-free signals and accurately detect spikes in diverse scenarios, thereby demonstrating the robust generalization capabilities of our stimulation artifacts rejection technique.

## 2. Methods

### 2.1. Rejection algorithm

The underlying rationale for logLIRA is to achieve effective decoupling between natural electrophysiological activity and stimulus artifacts, minimizing the likelihood of secondary or residual artifacts due to inadequate artifact suppression.

To successfully achieve this goal, our novel approach operates through a two-step process. Initially, the algorithm identifies and subtracts the shape of each individual artifact from the signal. Subsequently, the clean signal segments are organized based on their distinctive features. This involves the rejection of highly synchronized, time-locked activity occurring at the beginning of the segments. This step aims to eliminate secondary artifacts introduced by the piece-wise linear interpolation, which may lead to erroneously identified spikes.

#### 2.1.1. Stimulation artifact shape estimation

The logLIRA algorithm is tailored to handle each stimulation artifact individually, treating all channels independently and not making assumptions about other stimuli within the same channel. This approach is critical, as the shape of a stimulation artifact is significantly influenced by factors such as the relative positioning of the stimulation and recording electrodes, the prior activity history of the channel, grounding configurations, and other hardware-specific considerations [1, 20, 36].

**Figure 1.**
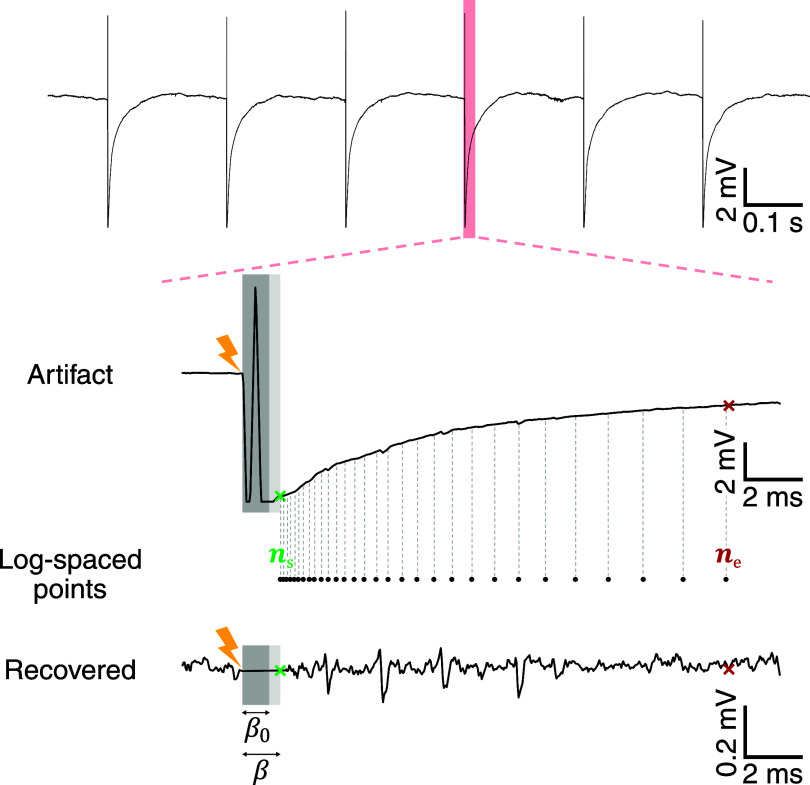
Representative stimulus artifact and its key points. The upper panel presents *K* subsequent stimulus artifacts, each characterized by similar profiles with individual variability and superimposed neural activity. The central row provides an enlarged view of the *i*th artifact, offering a detailed appreciation of its shape, highlighting the initial saturation of the signal. Logarithmically spaced interpolation points are used to estimate the artifact shape and then subtract it. The bottom panel represents the recovered signal segment, devoid of the suppressed stimulation artifact, enabling a clean view of the underlying neural activity (i.e. short-latency evoked spikes). The figure also indicates the stimulation onset, the default blanking period *β*_0_, and the full blanking period *β*. Additionally, the starting and ending recovery points (i.e. the interval where the estimated artifact is subtracted), denoted as *n*_s_ (green) and *n*_e_ (red) respectively, are reported on the exemplar artifact profile.

**Figure 2.**
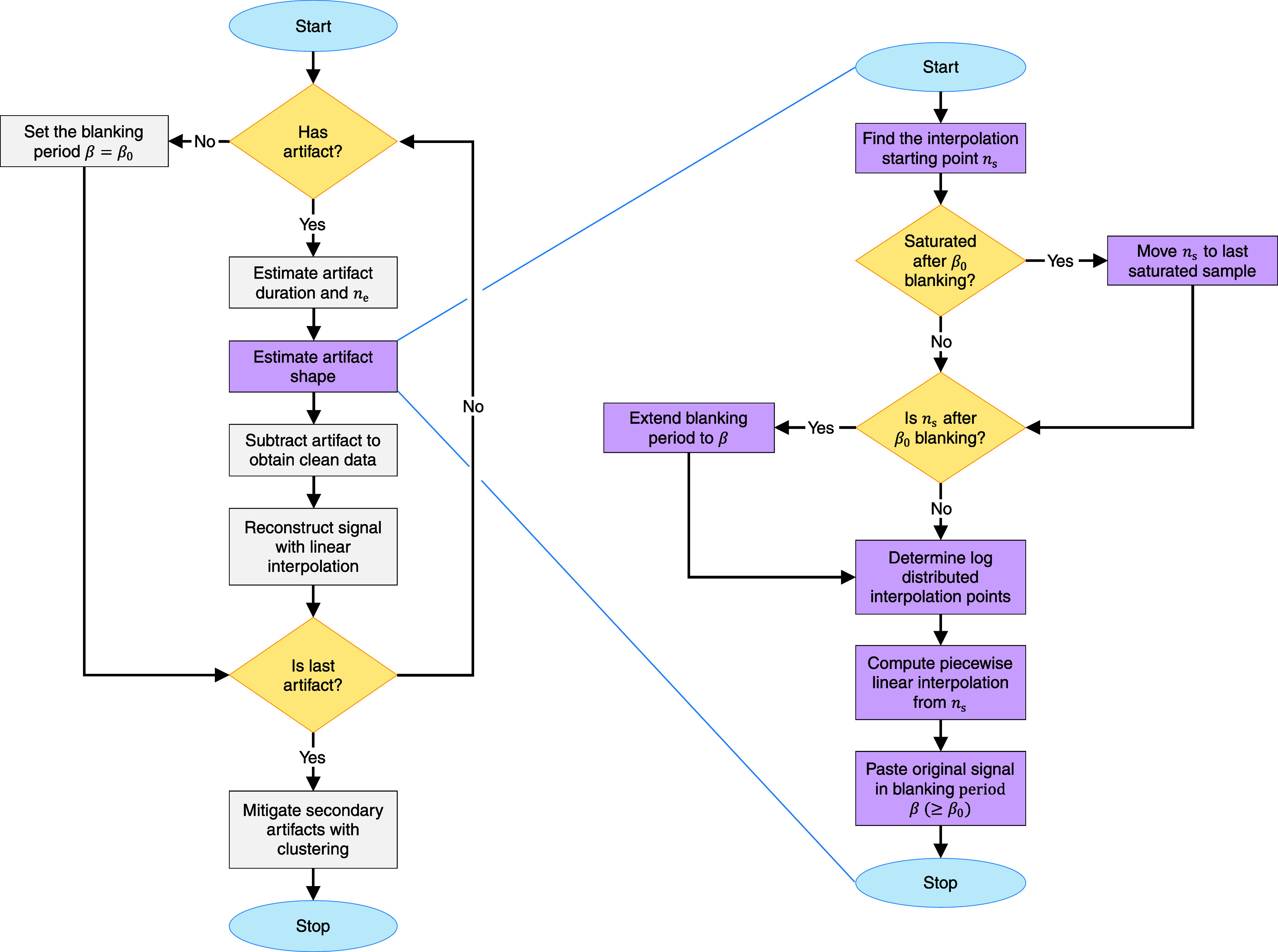
Flowchart describing the functioning of logLIRA. The diagram on the left side illustrates the algorithm from a macroscopic point of view. It displays that each stimulation artifact is addressed individually, estimating the artifact shape and subtracting it from the original signal segment. Eventually, clustering is performed on the initial portion of the clean segments to mitigate secondary artifacts, that are false positives arising from the cleaning process. The chart on the right is a detailed explanation of the stimulation artifact shape estimation through a piece-wise linear interpolation with logarithmically distributed interpolation points. *β*_0_, *β*, and *n*_s_ denote the initial blanking interval, the full blanking interval after dynamic extension, and the starting recovery point, respectively.

A signal typically presents a variable number of stimuli, here denoted as *K*. Hence, there will be *K* segments or trials of post stimuli recordings, each of possibly variable length *N*_*i*_, with $i = 1,\dots,K$ (see figure 1), equal to the inter-stimulus interval. In this time window, logLIRA identifies both the fast and slow artifact responses corresponding to the *i*th stimulus. The operations carried out by logLIRA are schematized in figure 2.

Each trial exhibits a time interval immediately following the stimulus onset marked by rapid voltage changes coinciding with the delivery of the stimulation electrical pulse. An illustration of this phenomenon is provided in figure 1. The initial portion of the to-be-recovered segment oscillates between the lower and upper voltage limits of the recording system, potentially causing temporary saturation of the acquisition system’s amplifier. Consequently, this initial fraction of each trial, often referred to as blanking interval, is considered unrecoverable and thus disregarded. The logLIRA algorithm relies on a predefined blanking period of fixed length, denoted by *β*_0_ and typically set at 1 ms. However, the algorithm is designed to autonomously extend *β*_0_ if it detects a prolonged intractable artifact portion, leading to the actual blanking period *β*, which is then utilized in the subsequent steps described below.

To minimize logLIRA intervention and unnecessary modifications of the recorded signal, each segment is examined to make sure it contains a significant artifact transient that corrupts the signal beyond the default blanking interval *β*_0_. This is done by comparing two windows of 5 ms each, before and after the blanking period: if the statistics (i.e. mean and standard deviation) of the two chunks of signal are similar enough, it is assumed that no transient is present and only the minimum blanking interval *β*_0_ is discarded. This step was introduced due to the observation that channels further away from the stimulation site often exhibit a negligible artifact transient.

If an artifact transient is indeed detected, the algorithm proceeds to estimate its duration so that the original recording is not edited unnecessarily. To accurately determine the artifact duration, the algorithm applies a 5 ms long moving average filter to the trial and subsequently compares the filtered signal to the median steady state value of the segment. The median is computed by considering all samples after a reasonable settling time (e.g. 40 ms) and the next stimulus onset. The first point where the filtered signal intersects the median below a $1\,\,\,\mu$V tolerance is detected as the transient’s end *n*_e_ (see figure 1). If the whole trial is shorter than the predefined settling time, *n*_e_ is the last sample of the trial.

Once the artifact duration is determined, the next step is to identify *n*_s_, the point from which the clean signal can be recovered (marked in green in figure 1). This corresponds to the first sample after the default blanking interval *β*_0_ (shown in dark gray) or, if saturation persists beyond *β*_0_, the first non-saturated sample. Saturation can be detected using a hard threshold set at 95% of the amplifier’s operating limit or, if that value is unknown, at 95% of the signal’s maximum voltage. When multiple signal segments exceed this threshold, they are concatenated. The blanking interval is then extended to *n*_s_ and denoted by *β* (shaded in light gray). The interpolation points are logarithmically spaced over time and later converted to samples to maintain consistency across systems with different sampling rates. Specifically, we can define a logarithmic grid made by *P* points between 0 ms and *T* − 1 ms (in milliseconds) as:

\begin{align*} t_j = 10^{\frac{j}{P-1}\cdot{\log_{10}\left(T\right)}}-1 \quad \mathrm{with}\;\; j = 0,\dots,P-1.\end{align*}

Subsequently, the interpolation points are transformed to samples by converting them to seconds and multiplying by the sampling rate *f_s_*:

\begin{align*} n_j = \mathrm{round}\left(t_j\cdot{10^{-3}\cdot{f_s}}\right) \quad \mathrm{with}\;\; j = 0,\dots,P-1.\end{align*}

The interpolation points are finally shifted by the blanking interval *β* to the right of the stimulation onset, and any point located beyond the recovery ending *n*_e_ is discarded. In this study, we set *P* = 42 and *T* = 50 ms. A visual representation of the interpolation points calculated using the above equations is shown in figure 1.

Given two generic interpolation points *n_j_* and $n_{j+1}$, a line is traced between their values $g(n_j)$ and $g(n_{j+1})$. The function $g(n_j)$ considers a variable size neighborhood *ε*, roughly proportional to the distance between *n_j_* and $n_{j+1}$, centered in *n_j_* and computes the mean value of the signal *s* in such neighborhood:

\begin{equation*} g\left(n_j\right) = \frac{1}{\epsilon+1}\sum_{k = -\epsilon/2}^{\epsilon/2}s\left(n_{j+k}\right).\end{equation*}

This process is repeated for all interpolation points $n_j\in{[n_{\mathrm{s}},n_{\mathrm{e}})}$. All lines are joined together and the blanked portion of the segment is pasted without modifications, leading to an accurate estimate of the stimulation artifact shape.

The retrieved artifact shape is then subtracted from the initial raw segment. To avoid discontinuities in the signal ($n_{\mathrm{e}}\unicode{x2A7D}{N_{i}}$) that might introduce ringing after bandpass filtering and cause secondary artifacts, a line is traced between the point preceding the stimulation onset and the point following *n*_e_. Then, the clean segment is superimposed on this line.

#### 2.1.2. Secondary artifacts mitigation

A piece-wise linear interpolation with logarithmically distributed interpolation points was used to approximate the signal immediately following the blanking interval *β*. However, this method may encounter challenges when dealing with rapid signal fluctuations within this region following amplifier saturation. As a result, the subtraction process between the original signal and the artifact estimate may inadvertently generate secondary artifacts, eventually leading to false positives or even signal corruption when subjected to bandpass filtering, due to the introduction of unwanted ripples. These secondary artifacts consistently appear in proximity to the blanking period across multiple trials, where the voltage changes are steeper (see figure 5 for an example of this phenomenon).

To address this issue, we considered a heuristically defined window of 2 ms immediately following the blanking interval *β* across all *K* trials. These signal chunks were grouped based on their characteristic shapes, with the goal of extracting and later subtracting the mean artifact shape per group. Due to the deterministic nature of logLIRA, secondary artifacts tend to be similar within a cluster, while the underlying neural activity is often less perfectly aligned across trials and is therefore attenuated by cluster averaging [37]. This generally yields a mostly activity-free secondary artifact estimate, although highly stereotyped short-latency evoked spikes may still leave a residual neural contribution, resulting in their partial attenuation. This strategy positions logLIRA as a hybrid between per-trial artifact correction methods and approaches that rely on averaging across trials to estimate artifacts.

Operationally, after the collection of the *K* signal chunks, the clustering step was carried out using uniform manifold approximation and projection (UMAP) with *distance* parameter set as *correlation*. This step projects the data into a two-dimensional space while preserving both local and global structure [38].

Finally, the low-dimensional representations of the 2 ms long signal chunks are clustered using density-based merging (DBM). This method exploits the local density of data points for clustering and does not require specifying the number of clusters *a priori* [39]. Clusters counting less members than a threshold *θ* = 20 are discarded. The surviving clusters are used to compute the mean secondary artifact shape, which is later subtracted from all trials in the cluster.

### 2.2. Synthetic dataset generation

**Figure 3.**
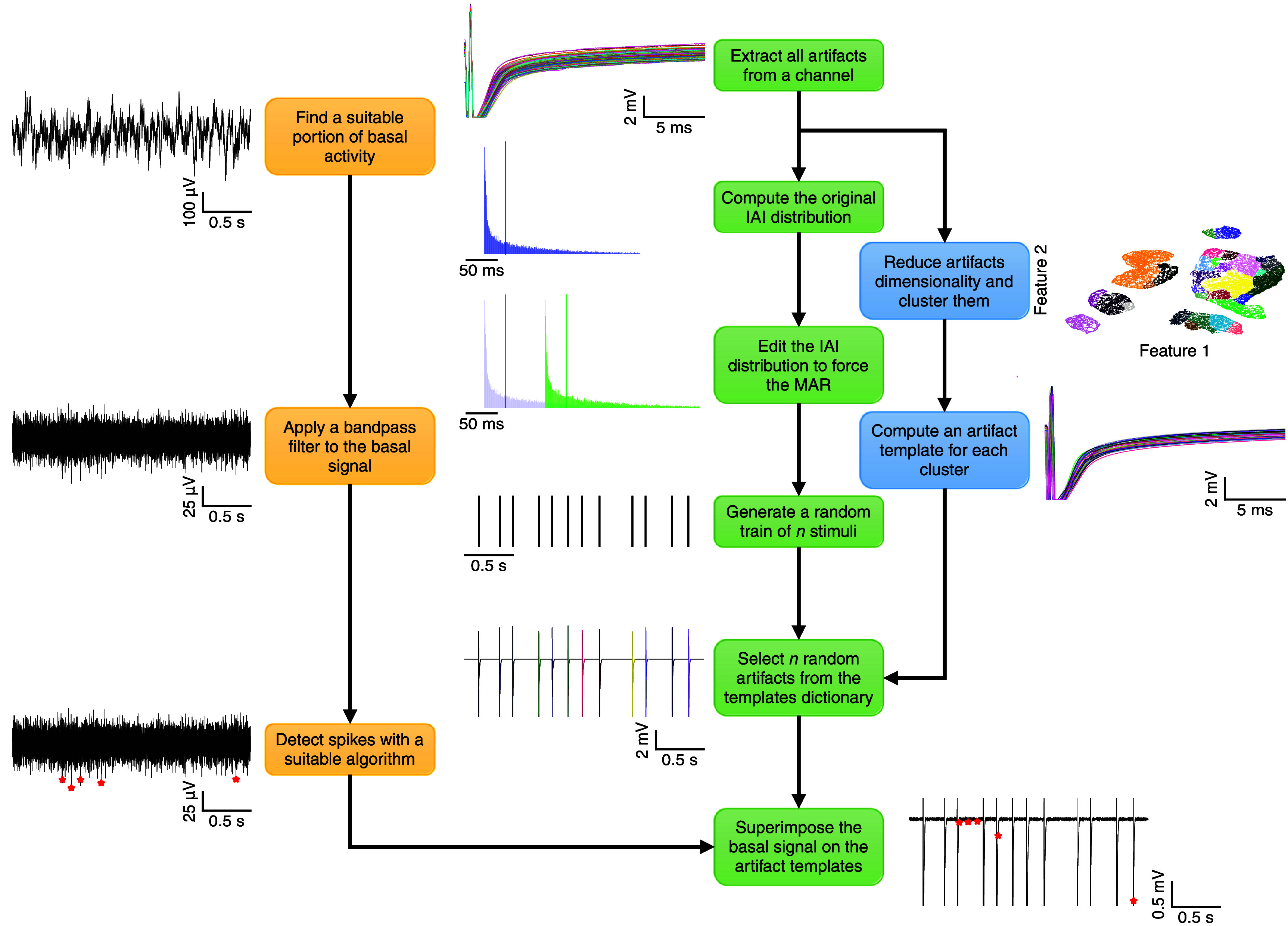
Semisynthetic benchmark dataset generation scheme. A block diagram illustrating the main steps involved in the synthesis of the dataset used to compare the performances among different stimulus artifacts rejection algorithms. All the blocks are accompanied by an example derived from *in vivo* rat recordings. The pipeline on the left (yellow) shows how the basal activity and the spike train acting as ground truth is generated starting from somatosensory (S1) cortex activity recorded while providing intracortical microstimulation following the activity-dependent stimulation (ADS) protocol. The central sequence of steps (green) illustrates the synthesis of an artificial train of stimuli with an inter-artifact interval (IAI) distribution resembling the original one. The right-most blocks (blue) describe the procedure used to extract the artifacts templates from the considered channel, later used to build a synthetic snippet that serves as a ground truth.

The creation of a synthetic dataset is pivotal for benchmarking the stimulus artifact rejection algorithms under consideration, as it provides a ground truth for comparison. However, it is equally important for the employed dataset to closely mirror the characteristics and properties of real data. Therefore, a data-driven approach to generate the dataset was employed (see figure 3), making it semisynthetic.

The generated dataset encompasses a predetermined range of possible mean firing rates (MFR $\in{\{5, 10, 15, 20, 25\}}$ Hz with a tolerance of $\pm{0.5}$ Hz) and mean artifacts rate (MAR $\in{\{2, 4, 6, 8, 10, 12, 14, 16, 18, 20\}}$ Hz with a tolerance of $\pm{0.1}$ Hz), defined by equation (4) as the reciprocal of the median of the inter-artifact interval (IAI). The selected MFR and MAR values are within the range of physiological values, especially for extracellular recordings carried out in the cortex, as was the case for the majority of the data used here. In addition, when imposing a certain MAR it is desirable to keep the initial distribution of IAIs consistent.

In order to assess the generalizability of the considered algorithms for the suppression of stimulus artifacts, it was imperative to build the semisynthetic benchmark dataset on top of electrophysiological data recorded under various conditions to enhance the diversity of the dataset. To this end, we have incorporated data coming from three distinct experiments not primarily intended for this work [33, 40, 41]. Our data were acquired from both Long–Evans rats (*Rattus norvegicus*) and squirrel monkeys (*Saimiri sciureus*), with unevenly spaced electrical stimuli delivered to the somatosensory cortical area and electrophysiological recordings acquired from neighboring cortical regions. Extensive descriptions of the experimental protocols are provided in the Supplementary Material.

Each one of the previously mentioned experimental groups is treated independently and constitutes a complete dataset on its own. The full dataset used as a benchmark is the combination of these individual subsets. The three sets contain 50 different snippets with a duration of 150 s each, covering all the possible combinations between the previously reported MFR and MAR values. Henceforth, the full benchmark dataset encompasses 150 semisynthetic snippets.

The pipeline leading to the generation of the benchmark dataset can be divided into three main steps (color-coded diagram blocks in figure 3): the extraction of 150 s long segments of basal activity, the crafting of dictionaries containing stimulus artifacts templates, that are average stimulus artifacts waveforms without the superimposed neural activity, and the synthesis of semisynthetic snippets mimicking segments of recordings containing stimulus artifacts and ground truth electrophysiological activity.

#### 2.2.1. Segments of basal activity

For eachof the three subsets mentioned above, 50 segments of basal activity (i.e. spontaneous electrophysiological activity) were collected. Henceforth, a subset contains 10 distinct segments of basal signal for each MFR value. These 150 s long chunks were obtained by randomly scraping the raw signals of recording sessions free from stimulation, in an iterative way. An efficient procedure to reject recording artifacts (e.g. movement) and individuate segments of basal activity with the desirable MFR values was developed and is described in the Supplementary Material.

These segments of artifact-free electrophysiological activity later served as the ground truth signal for evaluating the performance of the artifact rejection algorithms, both in terms of continuous voltage traces and detected spike trains.

#### 2.2.2. Dictionaries of artifact templates

To obtain a diverse set of stimulation artifact templates, we started with the assumption that each channel exhibits stimulation artifacts with different shapes due to its properties, particularly the relative position with respect to the stimulation site [20, 21]. However, individual channels were also characterized by a non-negligible internal variability of the stimulation artifacts [1]. In particular, the stimulation history (i.e. the distance between subsequent electrical pulses) affected the artifact waveforms, likely due to the changing operating conditions of the amplification system when a new stimulus was delivered.

Therefore, it was necessary to learn artifact templates on a per-channel basis, keeping the templates from different channels independent. Each collection of templates belonging to the same channel forms an artifact dictionary. Overall, each of our subsets, as defined above, contains 15 dictionaries of artifact templates, chosen and visually inspected to cover a diverse range of possible artifact shapes.

To build a dictionary for a specific channel, all the stimulation artifact waveforms are extracted and their length is selected according to the minimum IAI available. Then, the dimensionality of these waveforms is reduced by applying the UMAP method. This allows a bi-dimensional representation of all the channel’s artifacts that preserves both the local and global structure of data [38]. The features extracted from UMAP are then employed to group the artifacts according to their characteristics via the DBM clustering algorithm [39]. Clusters with less than 100 members are discarded, along with outliers detected by DBM. An artifact template for each accepted cluster is then obtained by computing the median between the group members and smoothing it with a lowpass filter (minimum-order FIR filter with a cutoff frequency of $\sim{2}$ kHz). This enables to capture only the dynamics related to the stimulus artifacts, discarding the underlying variable neural activity. Particular care is taken in treating artifacts exhibiting saturated periods, to prevent excessive smoothing during the transition between saturated and non-saturated regimes. In practice, the median-based template is combined with the previously mentioned lowpass-filtered version of the waveform, and the two are merged at the last time point where their difference exceeds 2.5 times its median value. This strategy preserves the fast onset transient and any saturation-related discontinuities, while suppressing slow oscillatory components caused by trial-to-trial neural variability. More details concerning the generation of artifact templates are provided in the Supplementary Material. All templates are eventually saved together with the channel’s train of stimulation onsets, constituting the dictionary of the stimulation artifact templates for that given channel.

#### 2.2.3. Snippets synthesis

The segments constituting the synthetic dataset, referred to as snippets, are generated by combining the component pieces obtained in the previous steps. Specifically, we generated 50 different snippets for each subset, such that the space defined by the MFR and MAR values is fully sampled.

The target or desired MAR can be computed as the reciprocal of the IAI median:

\begin{equation*} \mathrm{MAR} = \frac{1}{\mathrm{median}\left(\mathrm{IAI}\right)}.\end{equation*}

Therefore, to enforce the desired MAR, it is necessary to adjust the IAI accordingly. If the target MAR is smaller than the original one, then the IAI values can be easily shifted rightward, resulting in a shift of the IAI median towards larger values. Conversely, if the desired MAR is larger than the initial one, a small fraction of the largest IAI values is iteratively removed until the target MAR is reached. This results in a shrinkage of the IAI distribution, but the minimum IAI value is kept in place.

At this point, the modified IAI density function can be computed through kernel density estimation, to sample continuously distributed IAI values. After truncating it between the minimum and maximum IAI values, a new vector of IAIs is randomly extracted by means of an iterative approach until the total length of the snippet is reached. More in detail, by summing the obtained IAI values, the total snippet length is iteratively increased until the desired length (i.e. 150 s) is reached. Eventually, an artificial train of *K* stimuli can be immediately derived from the new IAI series.

For each stimulus, an artifact template is randomly picked from the dictionary associated with the corresponding channel. These *K* templates are then overlaid onto the snippet at time intervals dictated by the train of stimuli onsets, while the remaining sections of the snippet are set to zero. To avoid discontinuities following template insertion, each template is multiplied by a Blackman window. This process forces the template to decay to zero, ensuring seamless integration with the rest of the snippet. The exact shape of the window also modulates the artifact transient, increasing the decay speed. By varying the window shape, we introduce additional template variability. The full procedure is described in detail in the Supplementary Material.

Lastly, a basal signal with the desired MFR is randomly selected among the available ones and superimposed on the snippet, that now constitutes a ground truth since the spikes retrieved from the basal segment are known.

### 2.3. Benchmark analysis

To assess its performances, we tested logLIRA against a collection of previously reported algorithms that rely on different approaches. Specifically, we considered SALPA [20], which exploits local polynomial fitting and represents a well-established method to reject stimulation artifacts, a global polynomial fit method as implemented in [33], and dynamic averaging as described in [1], with minor modifications to enable unsupervised usage (see Supplementary Material). To ensure a fair comparison, we carefully selected the parameters for each algorithm based on methods’ documentation and preliminary testing on both real and semisynthetic data. While extensive and rigorous parameter sweeps were not performed, the chosen parameters represent a reasonable choice for optimal performance on our semisynthetic benchmark dataset (see supplementary table 2).

Despite their popularity, we excluded other notable stimulation artifacts suppression methods [18, 19, 35]. This choice was done due to incompatibilities between the algorithm assumptions (outlined in the Introduction section) and our semisynthetic dataset. Additionally, we also excluded filtering methods, as previous studies have highlighted lower performance compared to other considered approaches (i.e. SALPA) [20].

Properly defining the benchmarking process is a crucial step, as it allows a comparison of the output of a stimulus artifact rejection algorithm against a ground truth, quantifying how well such algorithm succeeds in suppressing stimulus artifacts while preserving the underlying neural activity. Importantly, the operations described below are repeated for each snippet belonging to the semisynthetic dataset introduced in the previous section.

First, the considered snippet is fed to the target algorithm, while the execution time is recorded. Since the returned output should ideally be equal to the basal activity segment superimposed on the artifacts during the snippet synthesis step, the algorithm’s output must go through the same type of processing. More precisely, the output is bandpass filtered with cutoff frequencies of 300 Hz and 7000 Hz using a 4th order zero-phase distortion Butterworth filter [42]. Subsequently, the basal signal and the output of the algorithm are subtracted from one another to compute the root-mean-square error (RMSE):

\begin{equation*} \mathrm{RMSE}_j = \sqrt{\frac{\sum_{i = 1}^{S}{\left(x_{i}-\hat{x}_{i}\right)^2}}{S}}\end{equation*}

where *S* refers to the number of samples available in the *j*th snippet, and *x*_*i*_ and $\hat{x}_{i}$ refer to the *i*th sample of the ground truth and the algorithm’s output, respectively.

At this point, the SWTTEO spike detection algorithm is applied to the filtered output [43].We selected the algorithm parameters to match the ones employed for the associated ground truth basal signal, reported in the supplementary table 1. Critically, SWTTEO is a deterministic algorithm: in an ideal case where the corrupted signal is perfectly recovered, we should expect a spike train exactly matching the ground truth one.

This step is followed by the classification of unmatched spikes; those either introduced (False Positives, FP), or disrupted (False Negatives, FN) by the stimulus rejection algorithm. These values are later normalized over the total number of ground truth spikes associated with the snippet taken into account. This step allows comparison of snippets with different MFRs. To account for slight differences in algorithmic reconstructions between methods, a jitter compensation window of $\pm{0.2}$ ms was introduced to compensate for negligible deviations in spike timing between ground truth and reconstructed signal.

To condense the information about false positives and false negatives in a single metric, we relied on *C*_0_, the cross-correlation at zero lag. In other words, *C*_0_ represents the degree of matching between the basal activity spike train, acting as a ground truth, and the spike train derived from the clean semisynthetic snippet, thus measuring the capability of a given stimulus artifacts rejection algorithm to maximize the recovery of the true underlying neural activity, minimizing the number of false positives.

Denoted by $C_{xy}(\tau)$ the cross-correlation between two generic spike trains *x* and *y* displaced by a custom lag *τ* according to [44–46], *C*_0_ is defined as:

\begin{align*} C_{0} = C_{xy}\left(0\right) = \frac{1}{\sqrt{N_{x}N_{y}}}\sum_{s = 1}^{N_{x}}{\sum_{t_{i} = -\frac{\Delta\tau}{2}}^{\frac{\Delta\tau}{2}}} x\left(t_{s}\right)y\left(t_{s}-t_{i}\right)\end{align*}

where *t*_*s*_ denotes the timing of an event in the *x* train, *N*_*x*_ and *N*_*y*_ are the total number of events in *x* and *y* respectively, hence *C*_0_ is constrained between 0 and 1. The $\Delta\tau$ term indicates the tolerance window and is set to 0.4 ms, accounting for both positive and negative jitters of 0.2 ms.

The benchmark analysis has been carried out via custom Matlab scripts executed on an HP Z4 G4 workstation equipped with 32 GB of RAM and an Intel Core i9-10 920X 3.50 GHz processor and running Windows 11.

### 2.4. Statistical analysis

Statistical analysis was performed in R (version 4.4.3, [47]) using linear mixed-effects models (lme4, [48]) and estimated marginal means (emmeans, [49]) for both *C*_0_ and RMSE. Our investigation focused not only on the absolute performance of the tested methods, but also on their dependence on features such as MFR and MAR. To account for potential distortion effects introduced by our testing procedure, data points were grouped according to both the injected artifact templates and the baseline signals used as ground truth.

For both metrics, the models took the following form:

\begin{align*} f\left(C_{0},\, \mathrm{RMSE}\right) &amp; \sim 1 + \mathrm{algorithm} \times \left(\mathrm{MAR} + \mathrm{MFR}\right) \nonumber \\ &amp; \quad + \left(1 + \mathrm{algorithm} \mid \mathrm{idBasal}\right) \nonumber \\ &amp; \quad + \left(1 + \mathrm{algorithm} \mid \mathrm{idTemplates}\right).\end{align*}

Here, $f(\cdot)$ is a strictly increasing transformation function employed to approximate Gaussianity in the data. Specifically, a logarithmic transformation (i.e. $\log(x)$) was applied to RMSE, while a Fisher *z*-transformation (i.e. $\mathrm{atanh}(x)$) was used for *C*_0_. Moreover, for the analysis of *C*_0_, only values greater than or equal to 0.5 were considered. This selection was made to mitigate a small yet significant bimodality in the data that interfered with model fitting. The observed bimodality was caused by dynamic averaging and global polynomial fitting disproportionately yielding *C*_0_ values below 0.5, as depicted in supplementary figure 5(a). This procedure artificially enhanced their resulting performance in the statistical comparison. See discussion for practical implications of this choice.

Model performance was evaluated using R’s performance library [50], and an overview of these results is available in the supplementary figures. Once a suitable linear model was identified, statistical inference was conducted on the estimated marginal means using the emmeans library [49]. Multiplicity correction was applied using Tukey’s Honest Significant Difference method, with full contrast tables provided in the Supplementary Material.

### 2.5. Qualitative analysis on real data

To further evaluate the practical applicability of logLIRA and its performance in real-world scenarios, we applied all algorithms to a selection of held-out recordings from the datasets used to build our semisynthetic benchmark (see Supplementary Material). This additional analysis was performed to test the stimulus artifact rejection algorithms in a scenario characterized by evoked spikes that are strictly dependent on the stimulation, a condition that was not matched in our semisynthetic dataset.

We analyzed recordings from individual channels obtained during experimental sessions involving activity-dependent stimulation [13], which delivered unevenly spaced biphasic pulses. Each recording was truncated after the first 5000 stimuli, and all artifact rejection algorithms were subsequently applied. Following preprocessing, spike detection was performed using the SWTTEO method [36] on the cleaned, bandpass-filtered signals, employing the parameters detailed in supplementary table 1.

In the absence of ground truth, we assessed each algorithm’s ability to recover plausible short-latency evoked responses by visualizing the detected spikes as raster plots aligned to stimulation onsets and by computing post-stimulus time histograms (PSTHs). These PSTHs summarize the cumulative neural response across multiple stimuli. Their shape and area together with the latency of the evoked spikes provide qualitative insights into the physiological plausibility of the recovered activity.

## 3. Results

**Figure 4.**
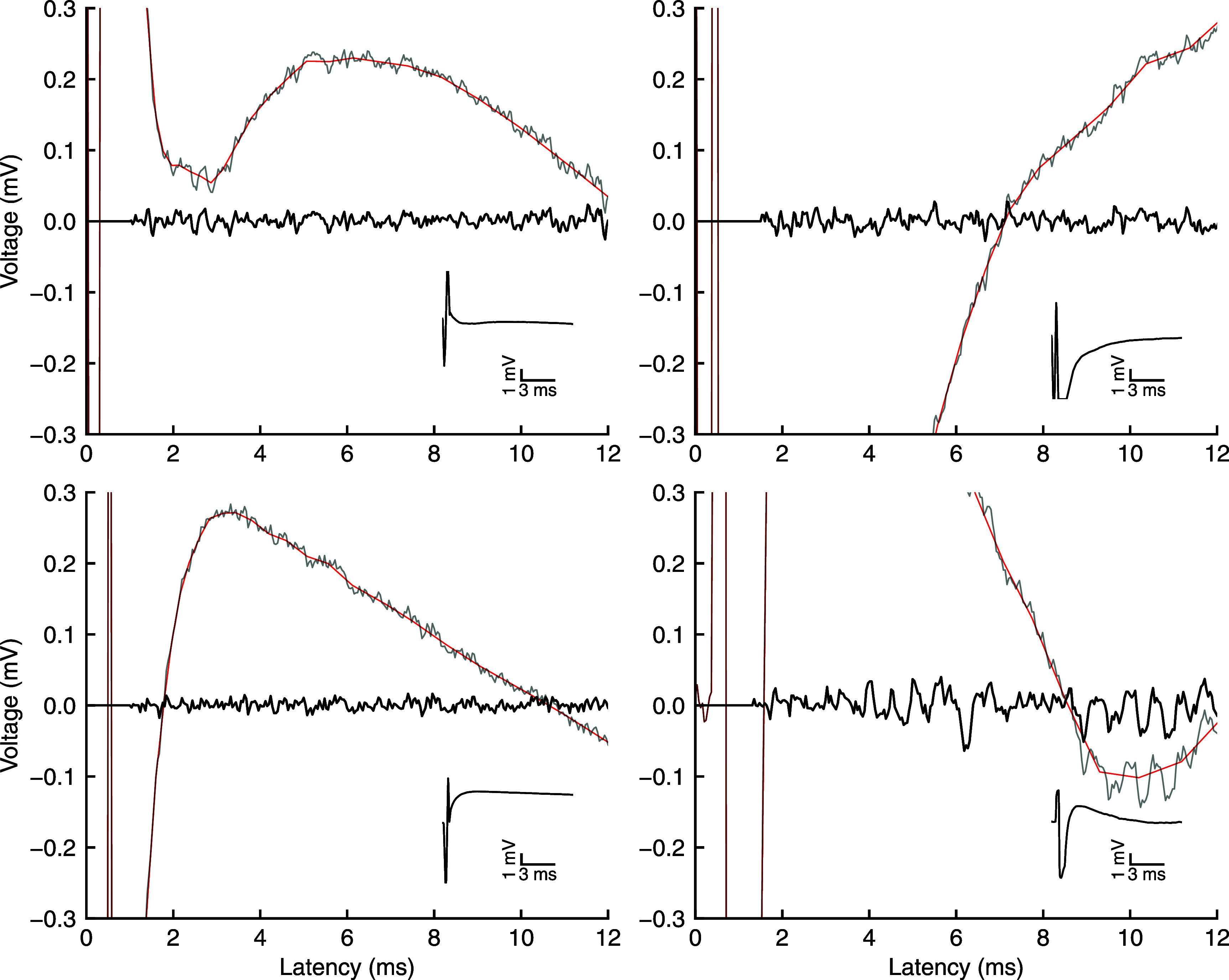
Artifact profile estimation and subtraction by logLIRA. For a variety of stimulation artifact waveforms (depicted in insets), logLIRA recovers activity even before 2 ms from the stimulus onset, also in presence of signal saturation. Faint traces represent the recorded data, where the artifact is present. A vertical offset is added for visualization purposes. Red lines are the artifacts estimated by logLIRA. Bold traces are the clean segments, before signal reconstruction and bandpass filtering.

To begin with, we qualitatively assessed the capabilities of our novel algorithm, logLIRA, in rejecting a variety of different artifact shapes minimizing the length of the blanking interval during the stimulus. Figure 4 clearly shows the capability of logLIRA to recover usable signal as early as 1 to 2 ms after the stimulus onset. Note that the length of the blanking period is dynamic and determined according to the features of the artifact itself. Our algorithm proved to be robust for different artifact shapes, including those affected by amplifier saturation, as depicted in figure 4 (see top-right panel for an example of amplifier saturation). The logarithmic spacing of interpolation points enables a good tracking of the signal even close to the stimulus delivery, mitigating secondary artifacts.

The following step consisted in comparing logLIRA to other widely used and affirmed methods to address stimulation artifacts. The most desirable feature for a stimulus artifact rejection algorithm, when specifically designed for short-latency evoked activity, is the ability to allow the detection of action potentials as close as possible to the stimulus onset. Moreover, it is critical to minimize the distortion of spike waveforms, as this might confound spike detection and sorting algorithms [20].

**Figure 5.**
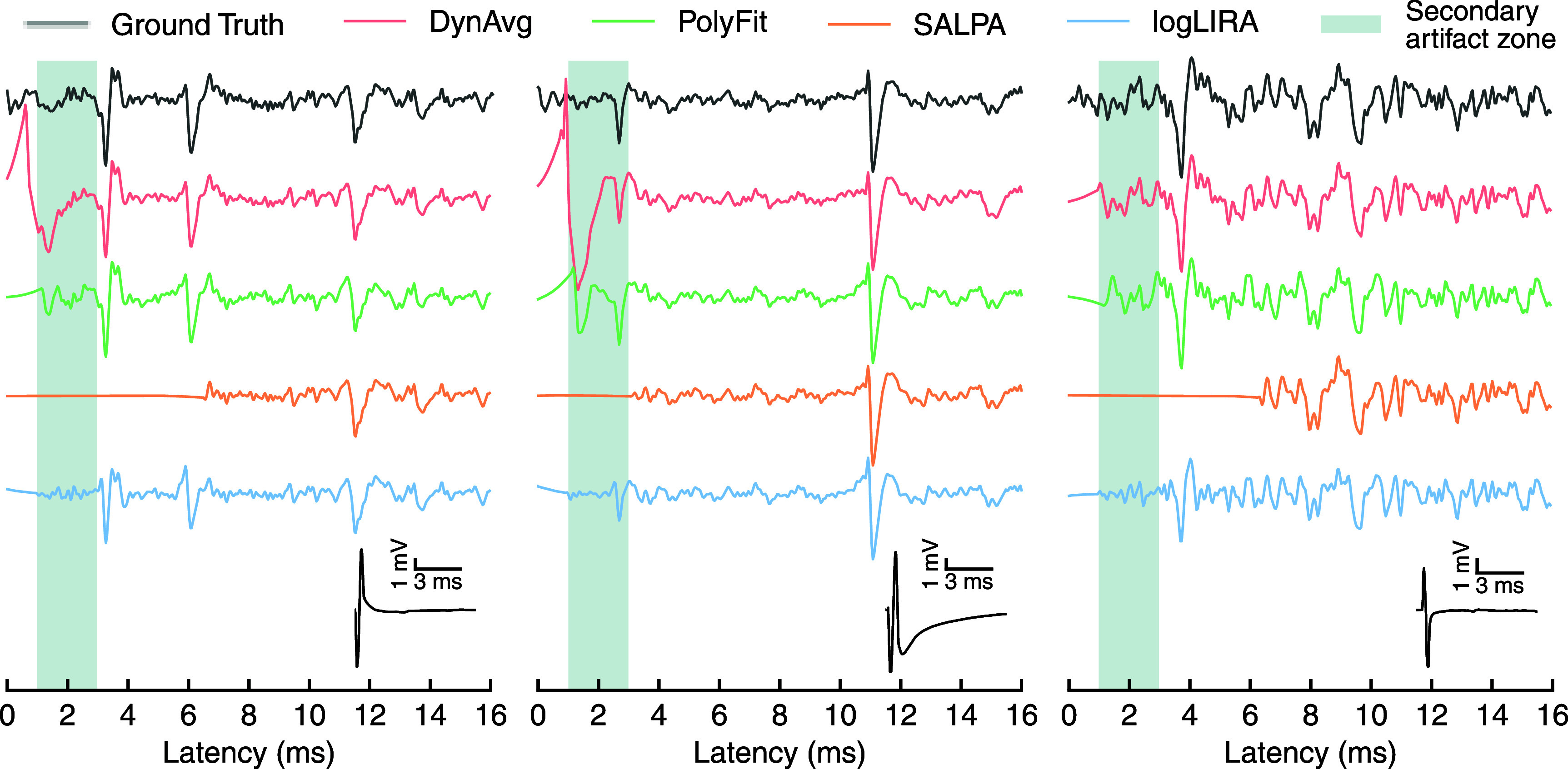
Comparison of the clean signal waveform among the considered algorithms. The figure shows three examples of signal immediately after a stimulus. The examples are obtained from synthetic snippets, hence a ground truth is available (black). The clean signals are the direct outputs from the corresponding stimulus artifact rejection algorithms, before any type of filtering is applied. An inset for each example displays the shape of the artifact corrupting the signal, highlighting the diverse shapes. A shaded region is also used to highlight the 2 ms long window following the blanking period, during which secondary artifacts (i.e. residuals of the artifact subtraction that might be mistakenly detected as spikes) are more likely to appear. Such residual artifacts can be noted in DynAvg and PolyFit traces, while SALPA is characterized by long blanking intervals hindering the observation of the short-latency response. logLIRA overcomes these issues, even though the first milliseconds of the recovered signal present a modest attenuation.

When it comes to the clean signal waveform, the method we propose here is capable to preserve the original shape of the signal, without modifying it significantly, as shown in figure 5. A non-negligible attenuation of the signal right after the blanking period can be observed. Nonetheless, it helps in the mitigation of secondary artifacts. Overall, SALPA seems to be the best choice when it comes to avoid any distortion in the signal waveform. However, it requires a substantially longer blanking interval *β*, hence it fails at perfectly tracking the signal at the beginning of the recovered segment. The polynomial fitting implementation we tested seems to produce acceptable results as well in terms of signal shape. Though, this technique is likely more prone to introduce secondary artifacts in the initial part of the recovered signal that may be detected as spikes. The last algorithm we tested, dynamic averaging, seems to fail in preserving the signal waveform, even if the low frequency oscillations it presents can be partially addressed via bandpass filtering.

We benchmarked the selected stimulus artifact rejection algorithms against our semisynthetic dataset as shown in table 1. The RMSE refers to the average distance between the ground truth signal and the recovered one, indirectly sampling also the ability of a stimulus artifact suppression algorithm in preserving the spike waveforms. Overall, logLIRA and SALPA outperform their competitors, with RMSE values slightly above $3\,\,\,\mu$V. When considering the fraction of mistakenly retrieved spikes (FP), logLIRA detects 3 times less false spikes than dynamic averaging and global polynomial fitting, while still overcoming SALPA, which false positive rate almost reaches 17%. Similarly, logLIRA and SALPA tend to miss approximately 10% of the ground truth spikes, which is 2.5 to 4 times less to what is overlooked by the other methods. The cross-correlation at zero lag *C*_0_ somehow summarize the FP and FN metrics, returning a value between 0 and 1 indicating how coincident two spike trains (i.e. the ground truth and the retrieved one) are. On average, logLIRA and SALPA are characterized by a *C*_0_ slightly smaller than 0.9, while global polynomial fitting mean *C*_0_ drops to 0.74. Similarly to what highlighted by FP and FN ratios, dynamic averaging performs worse than the other three techniques.

**Table 1.** Summary of the metrics computed to assess the performances of every tested algorithm. Results are reported as mean value and standard deviation. The root-mean-square error (RMSE) underlines the differences in the waveform between the ground truth and the reconstructed signal. It is expressed in *µ*V. The average time consumption is measured in ms to correct a single artifact. False negatives (FN) and false positives (FP) are reported as percentages over the true number of spikes. The cross-correlation at zero lag$(C_{0})$ measures the degree of matching between the baseline spike train, acting as a ground truth, and the spike train derived from the clean data. *C*_0_ is constrained between 0 and 1.

	RMSE $(\mu$V)	FP $(\%)$	FN $(\%)$	C_0_	Time Cons. (ms)
DynAvg	$15.49\pm19.13$	$36.05\pm44.84$	$41.27\pm33.71$	$0.60\pm0.34$	$\mathbf{0.70\pm0.17}$
PloyFit	$5.95\pm2.66$	$34.01\pm45.32$	$24.70\pm24.00$	$0.74\pm0.24$	$3.31\pm0.99$
SALPA	$3.65\pm1.85$	$16.99\pm18.45$	$10.10\pm4.96$	$0.88\pm0.08$	$1.67\pm2.05$
logLIRA	$\mathbf{3.26\pm1.47}$	$\mathbf{13.02\pm12.52}$	$\mathbf{9.57\pm6.15}$	$\mathbf{0.89\pm0.08}$	$3.51\pm4.73$

**Figure 6.**
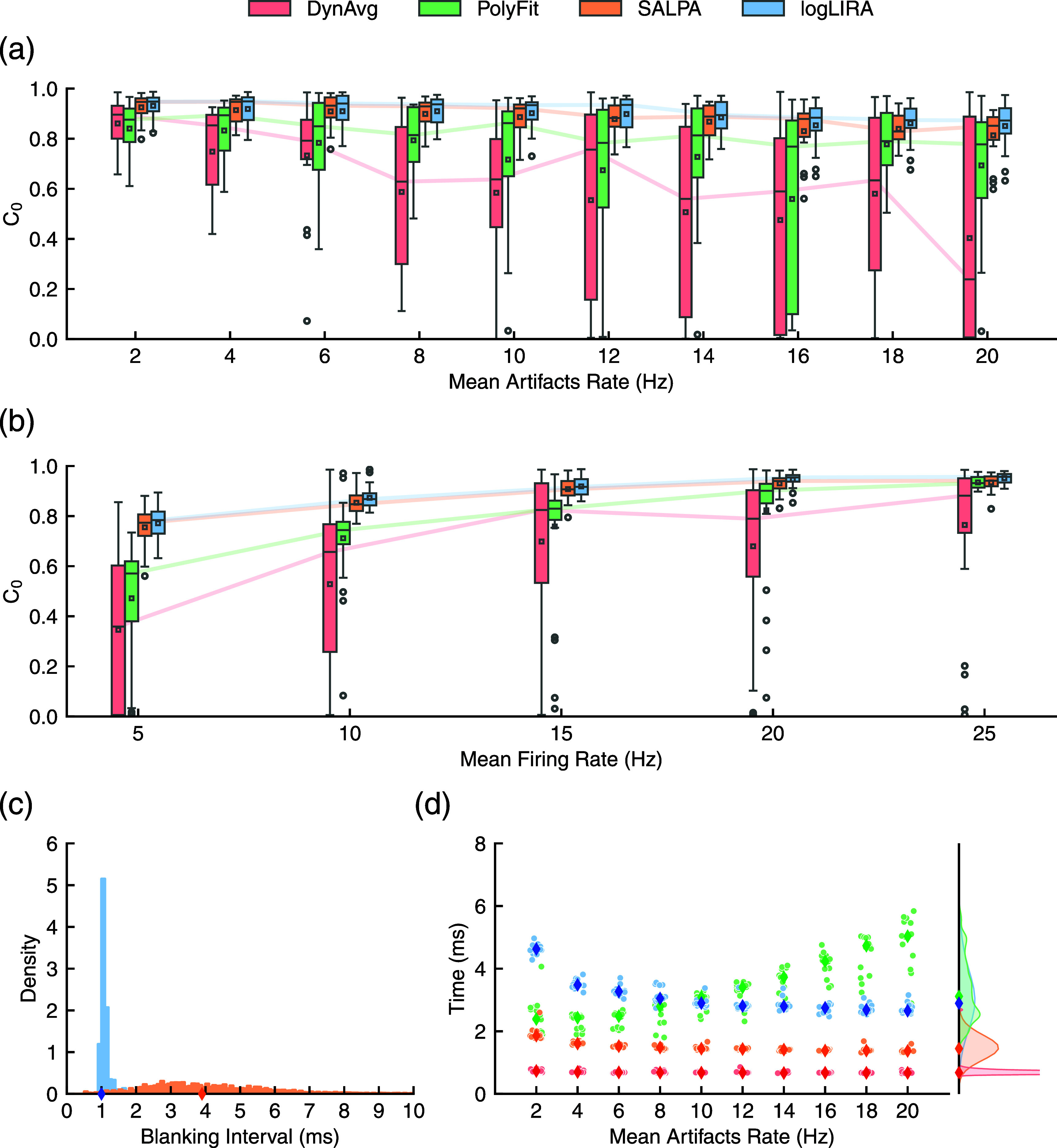
Quantitative comparison of stimulation artifact rejection methods. (a) The cross-correlation at zero lag *C*_0_ for each of the analyzed algorithms is grouped according to snippets mean artifact rate (MAR) values, that is the density of stimulation pulses. (b) Similarly, *C*_0_ values are grouped according to the snippets mean firing rate (MFR). (c) The distributions of the blanking intervals (i.e. signal completely lost after the stimulus) for logLIRA and SALPA, the best performing algorithms, are depicted as histograms. Median values are indicated by diamond-shaped markers. (d) Average time consumed by each tested algorithm to reject a single stimulus artifact, grouped by MAR. The global distributions are plotted on the right side panel. Median values are indicated by diamond-shaped markers.

Figure 6 provides a graphical visualization of the quantitative results. In particular, figure 6(a) depicts the *C*_0_ as the stimulation artifacts density increases for the four tested algorithms. The panel clearly shows that logLIRA and SALPA outperform global polynomial fitting and dynamic averaging. The former two methods exhibit a greater consistency in their performance regardless of the MAR, with a significantly smaller drop in the fraction of correctly identified spikes. The linear mixed-effects model fitted to the data (see supplementary figure 5 and supplementary table 5) indicates that for low MAR levels there is no statistical difference between logLIRA and SALPA. However, as the number of artifacts per unit of time grows, our novel algorithm consistently outperforms SALPA. By grouping the *C*_0_ values according to the snippets MFRs (see figure 6(b)), dynamic averaging proves to be less consistent than its competitors. Generally, logLIRA and SALPA are marked by similar performances. Global polynomial fitting is characterized instead by poor performances for low MFR values, though it seems to work very well when the overall number of ground truth spikes increases.

Similar results hold for RMSE, quantifying the distance between the recovered traces and the ground truth ones, presented in the supplementary figure 6. However, despite the overall difference between our algorithm and SALPA is small, it proved to be extremely consistent, always yielding a statistical difference between the two methods, as reported in supplementary tables 9 and 10. A key difference between SALPA and our newly introduced algorithm is summarized in figure 6(c). This panel depicts the distribution of average blanking intervals, thus the unrecoverable signal segments, for all the 150 semisynthetic snippets. logLIRA tends to discard only 1 ms of the recorded signal, while local polynomial fitting (SALPA) has a median blanking interval of 4 ms, leading to a larger loss in the original recorded signal. Eventually, figure 6(d) illustrates the average time consumed by each tested algorithm to suppress a single stimulus artifact. Data are grouped according to the MAR to highlight their performance as the number of artifacts per unit of time increases. Dynamic averaging is without surprise the fastest method, with a constant execution time below 1 ms per artifact. SALPA is particularly efficient as well, taking always no more than 2 ms to suppress a stimulus artifact. Global polynomial fitting and logLIRA exhibit different behaviors instead. The former requires more time as the artifacts’ density grows, up to approximately 5 ms. Conversely, logLIRA unitary execution time decreases for higher MAR values, approaching 3 ms when the median stimulation rate reaches 20 Hz.

**Figure 7.**
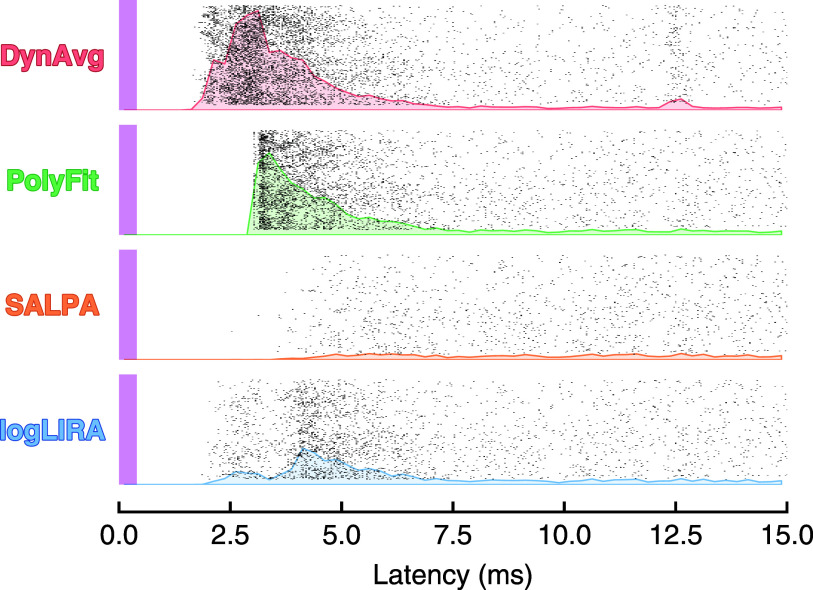
Recovered short-latency evoked activity from a real signal. Stimulation artifacts from a channel adjacent to the stimulation site were suppressed from 5000 consecutive trials by employing the stimulation artifact rejection algorithms considered in this work. Action potentials were detected using the stationary wavelet transform-based Teager energy operator (SWTTEO) after bandpass filtering. The purple bars represent time and duration of the stimuli. Post-stimulus time histogram (PSTH) plots are shown in the background for each tested artifact rejection technique with a bin width of 0.25 ms. Note the highly aligned activity recovered with PolyFit across almost all trials, not observed with the other methods. SALPA instead fails to recover any short-latency response due to its long blanking intervals.

Moving from semisynthetic to real data, another crucial qualitative insight on the performances of the tested stimulus artifact rejection algorithms is provided by the raster plots presented in figure 7. In real-world settings, with stimulation-evoked spiking activity, logLIRA captures a good short-latency response. On the contrary, SALPA is unable to recover usable signal earlier than approximately 4 ms from the stimulation onset. This results in a raster plot exhibiting no evoked activity. The algorithm exploiting polynomial fitting shows a suspicious extremely aligned response. It is localized slightly after 2.5 ms from the beginning of the stimulus. It is likely to be the consequence of time-locked secondary artifacts that are mistakenly identified as spikes, giving rise to false positives. Concerning the dynamic averaging method, it displays a strong short-latency evoked response. However, the subsequent activity appears to be slightly different compared to the one recorded by the other three algorithms.

## 4. Discussion

The rejection of electrical stimulation artifacts is a crucial step when investigating the neural circuitry response to externally provided electrical pulses. The wide variety of existing algorithms, each with its strengths and weaknesses, makes it challenging to determine the best approach for a given experimental setup. The preservation of the original signal and the detection of evoked neural activity are heavily dependent on the specific characteristics of the artifacts themselves, which are in turn informed by the experimental design choices, such as the recorded brain areas, the stimulation protocol, and the electrodes’ placement among others. Therefore, a comprehensive evaluation of different algorithms on a large and diverse dataset of semisynthetic snippets is essential to compare their capabilities in an unbiased way.

According to the values reported in table 1 and displayed in both figure 6 and supplementary figure 6, logLIRA and SALPA emerge as the most reliable methods, among those tested, in rejecting artifacts generated by electrical stimulation pulses, with almost no difference between them, considerably outperforming dynamic averaging and global polynomial fitting.

Interestingly, the poor performances of the latter two algorithms are highlighted by the average scores and by the heterogeneity in their performances (figure 6) reflected by the standard deviations reported in table 1. This suggests that dynamic averaging and global polynomial fitting do not handle equally well different types of stimulation artifacts, depending on their profiles or distribution over time. The poor performances of dynamic averaging in particular can be attributed also to the fact that in our synthetic dataset there is no relationship between subsequent artifact profiles, they were simply obtained from the same channel, but their temporal appearance is totally uncorrelated from their shape, making the estimation of an average artifact waveform unreliable [1]. logLIRA and SALPA obtained higher degrees of generalization, performing consistently across different types of artifacts, with a median *C*_0_ of around 0.9 for both methods.

Despite the high level of fidelity in the activity reconstructed by SALPA, the wide distribution of blanking periods observed in our tests (figure 6(c)) suggests that this method may not be well-suited to recover the short-latency evoked activity. The portion of signal fully blanked after a stimulus onset is highly variable, with a median value of almost 4 ms, often resulting in the irremediable loss of any monosynaptically evoked responses. This limitation is shown in both figures 5 and 7.

It is worth noting that the nature of our semisynthetic dataset cannot model evoked responses, as spikes are randomly scattered throughout the snippet length, originating from recording segments of basal activity. Therefore, the aforementioned limitation of SALPA is not readily reflected in *C*_0_ and *RMSE* metrics. We believe SALPA would be characterized by a higher false negative rate in real world application, due to its excessive blanking intervals, which can obscure evoked activity, as depicted in figure 7. Furthermore, because logLIRA is designed to preserve usable signal at very short latencies while limiting secondary artifacts, we expect its performance advantage over the other tested methods to extend to stimulus evoked activity, although our semisynthetic benchmark could not directly validate this case and highly time locked responses may still introduce a small residual component during the cluster average subtraction step. supplementary figure 2 illustrates representative secondary artifact templates and processed trials in real recordings, showing how the subtracted templates had limited practical impact on short-latency spike recovery. While this aspect of our semisynthetic dataset may represent a limitation of the present study, our primary focus was on accurately reproducing spike waveforms and assess their distortion, in order to rigorously evaluate the impact of the tested artifact removal techniques on spike detection and sorting performance.

A further limitation of the present study is that we did not evaluate protocol-centered artifact suppression strategies, such as forward masking or alternating polarity stimulation, which require dedicated acquisition paradigms rather than operating purely *post hoc* on recordings acquired under uniform stimulation conditions [28, 51, 52]. We omitted these approaches because our benchmark was designed to compare protocol-agnostic methods applicable to previously collected ICMS data. Moreover, our dataset highlights the challenges of transferring protocol-centered schemes to this domain: even within a single channel, stimulation artifacts exhibited non-negligible variability and strong dependence on stimulation history, particularly inter-pulse intervals, likely reflecting transient shifts in amplifier operating conditions. This variability motivated our choice to learn artifact templates on a per-channel basis and, more generally, logLIRA’s design principle of modeling each artifact individually rather than assuming a fixed transformation across trials. While protocol-centered approaches remain valuable for specific purposes, their assumptions must be verified explicitly when applied to ICMS recordings characterized by irregular pulse timing, variable artifact morphology, and potential acquisition system nonlinearities and nonstationarities.

More broadly, protocol-centered strategies are best suited for analyses centered on trial-averaged evoked responses, such as PSTHs. In our work, however, PSTHs were used only as a qualitative reference in the absence of ground truth, whereas the semisynthetic benchmark itself assessed waveform preservation and spike detection fidelity. In many ICMS applications, however, the scientific objective extends beyond the mean evoked response to encompass single-trial latency, response variability, and stimulation-evoked network dynamics [34, 53–55]. This distinction is particularly relevant in cortex, where variability in evoked responses and patterns of network entrainment are themselves informative [33, 55–57]. Because forward masking and alternating polarity paradigms estimate neural activity by combining trials obtained under modified stimulation conditions, they may inadvertently perturb the circuit state of interest. We therefore view these approaches as complementary tools to investigate specific experimental questions rather than substitutes for protocol-agnostic *post hoc* suppression. Importantly, this distinction also frames the motivation for methods like logLIRA, whose design goals emphasize faithful recovery of single-trial activity under predefined stimulation conditions.

To optimize the recovery of artifact-disrupted signal and minimize missed evoked spikes, logLIRA is designed to keep blanking intervals as short as possible. As showcased in figure 6(c), the blanking interval is reduced to a minimum, increasing its consistency and leading to a median value as small as 1 ms. This result is achieved by adding a second cascaded step responsible to treat secondary artifacts, often a consequence of excessively short blanking periods, enabling our algorithm not only to reduce the lost signal portions, but simultaneously keep under control the false positive rate otherwise inflated by the presence of secondary artifacts, a phenomenon clearly visible in real recordings (see figure 7).

We acknowledge that the secondary artifact mitigation stage of logLIRA employs UMAP for dimensionality reduction and DBM for clustering. The semisynthetic benchmark dataset also relies on these techniques when constructing artifact templates, raising the possibility of methodological overlap between benchmark generation and stimulation artifact suppression. However, the use of UMAP and DBM in the two contexts differs fundamentally in both purpose and data structure. In the semisynthetic pipeline, these methods are applied to full-length raw trials to cluster stimulation artifacts with similar waveform morphology, enabling the creation of representative template classes. By contrast, logLIRA applies UMAP and DBM to brief, partially cleaned short-latency (i.e. 2 ms) segments extracted immediately after $n_\mathrm{s}$, focusing specifically on capturing residual secondary artifacts. Because the informational content of the clustered data differ substantially between these two applications, we believe that the use of similar computational tools does not introduce circularity or systematically bias the benchmark in favor of logLIRA, ensuring a fair comparison among the tested algorithms.

It is important to note that the secondary artifact mitigation step is not present in the polynomial fitting algorithm we tested. Although the blanking period is even shorter than logLIRA (see figure 5), the lack of a secondary artifact handling mechanism can lead to a higher false positive rate. This is reflected in the results shown in table 1 and figure 6. In fact, the polynomial fitting algorithm is prone to introducing artifact profiles that are mistakenly identified as spikes, which are consistently present at the beginning of each trial, resulting in suspiciously aligned short-latency responses. This is visible in figure 7 obtained from real data.

Mitigating secondary artifacts introduces a trade-off for logLIRA. This process involves clustering partially cleaned signal segments that follow the stimulus onset and subtracting shared time-locked activity from each cluster. As a result, a sufficient number of trials is required to accurately capture the entire population and facilitate effective grouping of these segments. Our approach is less suitable when only a few electrical pulses are delivered. In principle, at least 20 stimulation artifacts are required for secondary artifact mitigation if the artifact shapes are highly consistent across trials and can be clustered together (see *θ* in section 2.1.2), though this represents a lower bound. In practice, greater variability in artifact waveforms demands more examples for accurate estimation. For typical use cases, a few hundred examples are generally sufficient to achieve reliable performance.

Additionally, logLIRA is not designed to support real-time stimulation artifacts suppression due to the above issue. Nonetheless, the computational burden of about 3.5 ms for the correction of a single artifact would not prevent us from applying the method online, as shown in table 1. The algorithm’s most computationally intensive steps, namely the fitting and subtraction of stimulation artifacts as well as the mitigation of secondary artifacts, exhibit sublinear scaling with increasing average stimulation rates (see supplementary figure 7). A viable workaround to adapt our algorithm to real-time settings might consist in collecting a large-enough set of partially cleaned signal fragments to build secondary artifact templates beforehand, assigning every new trial to an existing cluster and then removing the shared time-locked activity from it.

It is worth noting that our innovative algorithm is tailored for low to medium density electrophysiological recordings, where each channel is handled independently for both stimulation artifact rejection and spike detection or sorting procedures. For high-density recordings, alternative approaches that consider the integration of multiple adjacent channels may yield superior outcomes, especially when it comes to false positives’ rejection.

Despite its applicability limitations, logLIRA has proven to be a reliable and effective tool for the suppression of stimulation artifacts, successfully allowing for the recovery of short-latency evoked activity.

## 5. Conclusion

The development of logLIRA marks a significant advancement in the field of stimulus artifact rejection. By accurately recovering short-latency evoked activity while minimizing false positives and signal loss even under challenging conditions like saturation, logLIRA offers significant improvements over existing methods. This capability opens up exciting new opportunities for neuroscience research, allowing the ability to recover monosynaptically evoked responses to stimulation. logLIRA could, for instance, improve the estimation of mesoscale effective connectivity by leveraging the stimulation-evoked effective connectivity metric [33]. It also holds potential to advance research on stimulation-induced functional reorganization by exploring the dependency of evoked responses on the stimulation protocol [58]. This promising direction could enhance neuroprosthetic systems designed to treat focal brain injuries, thereby supporting patients in regaining independence and improving their quality of life [3, 13, 14, 59].

## Data Availability

The data that support the findings of this study will be openly available following an embargo at the following URL/DOI: https://doi.org/10.5281/zenodo.16 642 111 [61].

## Code availability statement

The source code for logLIRA is publicly available at https://github.com/barbaLab/logLIRA. The Matlab scripts to generate a semisynthetic dataset from raw data and to run the benchmark analysis are available at https://github.com/barbaLab/logLIRA-benchmark.
